# Rapid weather changes are associated with daily hospital visitors for atrial fibrillation accompanied by abnormal ECG repolarization: a case-crossover study

**DOI:** 10.1186/s40001-023-01632-3

**Published:** 2024-01-20

**Authors:** Shanmei Wu, Jingyi Guo, Xin Chen, Jie Wang, Gang Zhao, Shixin Ma, Tianzheng Hao, Jianguo Tan, Yongguang Li

**Affiliations:** 1 Shanghai Jiao Tong University School of Medicine, Shanghai, People’s Republic of China; 2 Clinical Research Center, Shanghai Sixth People’s Hospital Affiliated to Shanghai Jiao Tong University School of Medicine, Shanghai, People’s Republic of China; 3 Department of Cardiology, Shanghai Sixth People’s Hospital Affiliated to Shanghai Jiao Tong University School of Medicine, Shanghai, 200233 People’s Republic of China; 4 Department of General Surgery, Shanghai Sixth People’s Hospital Affiliated to Shanghai Jiao Tong University School of Medicine, Shanghai, People’s Republic of China; 5 Shanghai Meteorological IT Support Center, Shanghai, People’s Republic of China; 6 Key Laboratory of Urban Meteorology, China Meteorological Administration, Beijing, People’s Republic of China

**Keywords:** Meteorological factors, Atrial fibrillation, ECG, ST-T changes

## Abstract

**Background:**

Atrial fibrillation (AF) is highly prevalent in the population, yet the factors contributing to AF events in susceptible individuals remain partially understood. The potential relationship between meteorological factors and AF, particularly with abnormal electrocardiograph (ECG) repolarization, has not been adequately studied. This case-crossover study aims to investigate the association between meteorological factors and daily hospital visits for AF with abnormal ECG repolarization in Shanghai, China.

**Methods:**

The study cohort comprised 10,325 patients with ECG-confirmed AF who sought treatment at Shanghai Sixth People's Hospital between 2015 and 2018. Meteorological and air pollutant concentration data were matched with the patient records. Using a case-crossover design, we analyzed the association between meteorological factors and the daily count of hospital visitors for AF with abnormal ECG repolarization at our AF center. Lag analysis models were applied to examine the temporal relationship between meteorological factors and AF events.

**Results:**

The analysis revealed statistically significant associations between AF occurrence and specific meteorological factors. AF events were significantly associated with average atmospheric pressure (lag 0 day, OR 0.9901, 95% CI 0.9825–0.9977, *P* < 0.05), average temperature (lag 1 day, OR 0.9890, 95% CI 0.9789–0.9992, *P* < 0.05), daily pressure range (lag 7 days, OR 1.0195, 95% CI 1.0079–1.0312, *P* < 0.01), and daily temperature range (lag 5 days, OR 1.0208, 95% CI 1.0087–1.0331, *P* < 0.01). Moreover, a significant correlation was observed between daily pressure range and daily temperature range with AF patients, particularly those with abnormal ECG repolarization, as evident in the case-crossover analysis.

**Conclusion:**

This study highlights a significant correlation between meteorological factors and daily hospital visits for AF accompanied by abnormal ECG repolarization in Shanghai, China. In addition, AF patients with abnormal ECG repolarization were found to be more vulnerable to rapid daily changes in pressure and temperature compared to AF patients without such repolarization abnormalities.

**Supplementary Information:**

The online version contains supplementary material available at 10.1186/s40001-023-01632-3.

## Introduction

Atrial fibrillation (AF) is the most prevalent sustained cardiac arrhythmia, posing a significant threat to human health, especially with the aging population, which has led to a rise in its incidence and prevalence worldwide [1]. Some AF patients also exhibit abnormal electrocardiograph (ECG) repolarization, specifically manifesting as ST-T changes [2]. The ST-T segment on an ECG represents a period of no potential change between ventricular depolarization and repolarization, and any deviation from the normal pattern can indicate abnormalities [3, 4]. Studies have demonstrated that ST-T segment changes can serve as early warning signs of cardiovascular diseases [3, 5–8], making it crucial to investigate factors associated with these changes.

Previous research has shown that pollutants and meteorological conditions are associated with the incidence of AF [9–13]. For instance, a meta-analysis revealed significant associations between AF development and certain gaseous pollutants such as nitrogen monoxide (NO), carbon monoxide (CO), sulfur dioxide (SO_2_), ozone (O_3_), nitrogen dioxide (NO_2_), and particulate matter (PM) [14]. Increased ambient O_3_ has also been linked to paroxysmal AF [15]. Temperature and barometric pressure have been identified as contributing factors to AF events, with AF incidence being lowest in summer [16]. Moreover, studies have suggested that air pollution may affect cardiac rate and rhythm, potentially explaining the increased AF events observed on days with higher air pollution levels [17]. Overall, meteorological factors and environmental conditions have emerged as potential risk factors for AF [18, 19]. However, the specific relationship between meteorological factors and AF with ST-T changes has not been explored yet.

In this study, we aim to evaluate the association between meteorological factors and AF, particularly those exhibiting ECG ST-T changes. The meteorological factors considered include average atmospheric pressure (hPa), daily pressure range (hPa), average temperature (°C), daily temperature range (°C), average relative humidity (%), and average wind speed (m/s). We will use a case-crossover model to assess the risk of AF with ECG on different days, exploring the relationship between meteorological factors and daily hospital visits for AF with ECG ST-T changes.

By conducting this investigation, we hope to shed light on the potential influence of meteorological conditions on AF occurrence, specifically focusing on AF patients with abnormal ECG repolarization. Understanding these associations can aid in developing preventive strategies and early warning systems for cardiac episodes, ultimately contributing to better management and care for AF patients.

## Methods

### Study population

**Fig. 1:**
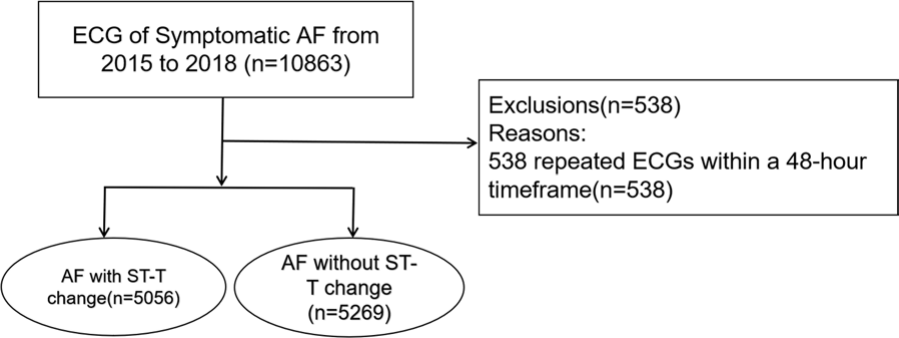
This flow chart represents the source process of patient data

AF, coding I48.x01 in 10th edition of International Classifcation of Diseases (ICD-10), was recorded by ECG. Generally, the ECG changes of typical AF are characterized by irregularly irregular R–R intervals, absence of distinct repeating P waves, and irregular atrial activation (ESC guidelines defnition). Patients’ data were eligible if they were testifed with ECG indicating AF from Jan 1st 2015 to Dec 31st 2018 in Shanghai Sixth People’s Hospital (Fig. 1). The ECG ST-T changes is defined as: the measurement of ST segment is based on TP segment, and the measurement points of ST segment are based on 60–80 ms after J point. ST segment elevation in normal people: limb leads less than 1 mm, chest leads less than 3 mm, V_1_–V_3_ less than 3 mm, V_4_–V_6_ less than 1 mm. Exceeding this range is regarded as abnormal. ST segment depression includes morphology and amplitude. Morphology: calculated at the angle between the vertical line of the R wave and the extension line of the ST segment, > 90° is a downward sloping type, = 90° is a horizontal type, and < 90° is an upward sloping type. Horizontal or downward sloping type of ST segment depression greater than or equal to 0.5 mm has pathological significance. T wave: T waves in leads R wave are upright, with an amplitude greater than 1/10 of the same lead R wave. T waves in leads III are upright and low-level inverted, while in leads AVF, they are upright and low-level. If T waves in leads V_1_–V_2_ are upright, T waves in leads V_3_–V_6_ cannot be inverted. If T waves in leads V_1_–V_2_ are inverted, T waves in leads V_3_ can be low and flat, and T waves in leads V_4_–V_6_ cannot be inverted [20, 21]. Our investigation aimed to assess the influence of meteorological factors on the daily hospital visits for AF patients indicated by ECG. Exclusion criteria involved excess records of AF with repeated ECG within a 48-h timeframe. The AF without ECG examination population will not be included in this study if their condition is stable or silent with AF, or if they are only undergoing follow-up visits without complete ECG (Fig. 1). Collected additional data included patient names, gender, age, and ECG information. Patients were divided into two groups based on the presence or absence of ST-T segment changes, and AF cases in these groups were analyzed concerning different meteorological factors (Table 1). To ensure consistency in AF classification, regular meetings were conducted. We are fully committed to respecting and safeguarding patient privacy, and the author assumes complete responsibility for the integrity of the data. All authors have reviewed and agreed to the manuscript.

**Table 1:** The basic characteristics of study population

Variables	AF (10325)	AF with ST-T change (5056)	AF without ST-T change (5269)	*P*-value
Male	47.78% (4933)	37.58% (1900)	57.56% (3033)	< 0.0001
Age, years	79 (69, 85)	80 (72, 85)	77 (68, 84)	< 0.0001
FVR	31.60% (3263)	32.59% (1648)	30.65% (1615)	0.0337
SVR	3.18% (328)	3.74% (189)	2.64% (139)	0.0014
CB	20.84% (2152)	16.06% (812)	25.43% (1340)	< 0.0001
NSVT	0.38% (39)	0.40% (20)	0.36% (19)	0.7721
VPB	12.31% (1271)	13.15% (665)	11.50% (606)	0.0107

Continuous variables were presented as median with interquartile range (M, IQR); Categorical variables were presented as *n* (%)

We collected information on ECG changes, including ST-T changes, fast ventricular rate (FVR), slow ventricular rate (SVR), conduction block (CB), nonpersistent supraventricular tachycardia (NSVT), and ventricular premature beat (VPB) (Table 1). On an electrocardiogram, the visual feature of AF is the disappearance of the P wave or the presence of the f wave, accompanied by an irregular time interval between QRS complexes, caused by irregular atrial pulse activation of the atrioventricular node [22]. The collected ECG data recorded the presence of ST-T changes, ventricular premature beats, ventricular tachycardia, conduction blocks, QRS, and other factors [23]. A comprehensive database was established through data collection and collation to facilitate a detailed analysis of AF with ST-T changes. This case-crossover study involving human participants adhered to the ethical standards of the institutional and national research committee, following the 1964 Helsinki Declaration and its subsequent amendments or comparable ethical standards. The study was approved by the ethics committee of Shanghai Sixth People’s Hospital.2.2.

We analyze the time series of the visit time of the patient and the meteorological factors (average atmospheric pressure, average temperature, average relative humidity, average wind speed, daily atmospheric pressure range, daily atmospheric temperature range), and get the time series diagram by combining the meteorological factors with correcting patient counts.

Weather data, including average atmospheric pressure (hPa), daily atmospheric pressure range (hPa), average temperature (°C), daily atmospheric temperature range (°C), average relative humidity (%), and average wind speed (m/s), were obtained from the Shanghai Meteorological Service. The daily averaged concentrations in Shanghai during the study period for fine particle mass and gaseous air pollutant were obtained to reduce the error and correct the results. From Shanghai Environmental Monitoring Center, PM_2.5_ (fine particle masses with diameter less than 2.5 μm, %), PM_10_ (fine particle mass with diameter less than 10 μm, %), O_3_ (ozone, %), SO_2_ (sulfur dioxide, %), NO_2_ (nitrogen dioxide, %), and CO (carbon monoxide, %) were recorded in Shanghai.

### Statistical analysis

Time-stratified case-crossover design, which is superior in controlling confounders of individual characteristics and can exclude long-term impact, was adopted to evaluate the associations between atmospheric pressure, atmospheric temperature, humidity, and the wind speed with AF with ECG. The interval between weather changes and AF occurrences with ECG confirmation is referred to as the lag time. The analysis covered the entire study population of AF patients with ECG. We continuously assessed AF risks related to meteorological factors at lag 0, lag 1, lag 2, lag 3, lag 4, lag 5, lag 6, and lag 7. To evaluate the lag structure, an unconstrained distributed lag model was used, measuring the number of days for each lag [24]. To identify any statistically significant relationship between meteorological factors and AF, we assessed the AF risk in relation to quartiles of each air pollutant (PM_2.5_, PM_10_, O_3_, SO_2_, NO_2_, and CO). The association was presented as an odds ratio (OR) with a 95% confidence interval (CI) for concentrations during lag 0–7 and daily hospital visits for AF, confirmed by ECG. This analysis was conducted using R (version 4.1.1; R Development Core Team, Vienna, Austria) with the season package, following the parameters defined in previous research [25]. Demographic distribution between the two groups was compared using Chi-squared tests and Mann–Whitney *U* test when appropriate. The association between air pollutant and meteorological factor was analyzed using the Spearman's Rho-related technology. To ascertain the robustness of the results, we conducted several sensitivity analyses. First, we removed the air pollutant variable from the main analytical model, and second, we used a distributed lag nonlinear model (DLNM) to capture the non-linear relation between the meteorological factors and the frequency of AF. Statistical significance was considered at *P* < 0.05 for all tests. All statistical analyses were conducted using the SPSS statistical package (version 20.0, SPSS Inc., Chicago, Illinois, USA).

## Results

### The basic characteristics of study population and meteorological factors

**Fig. 2:**
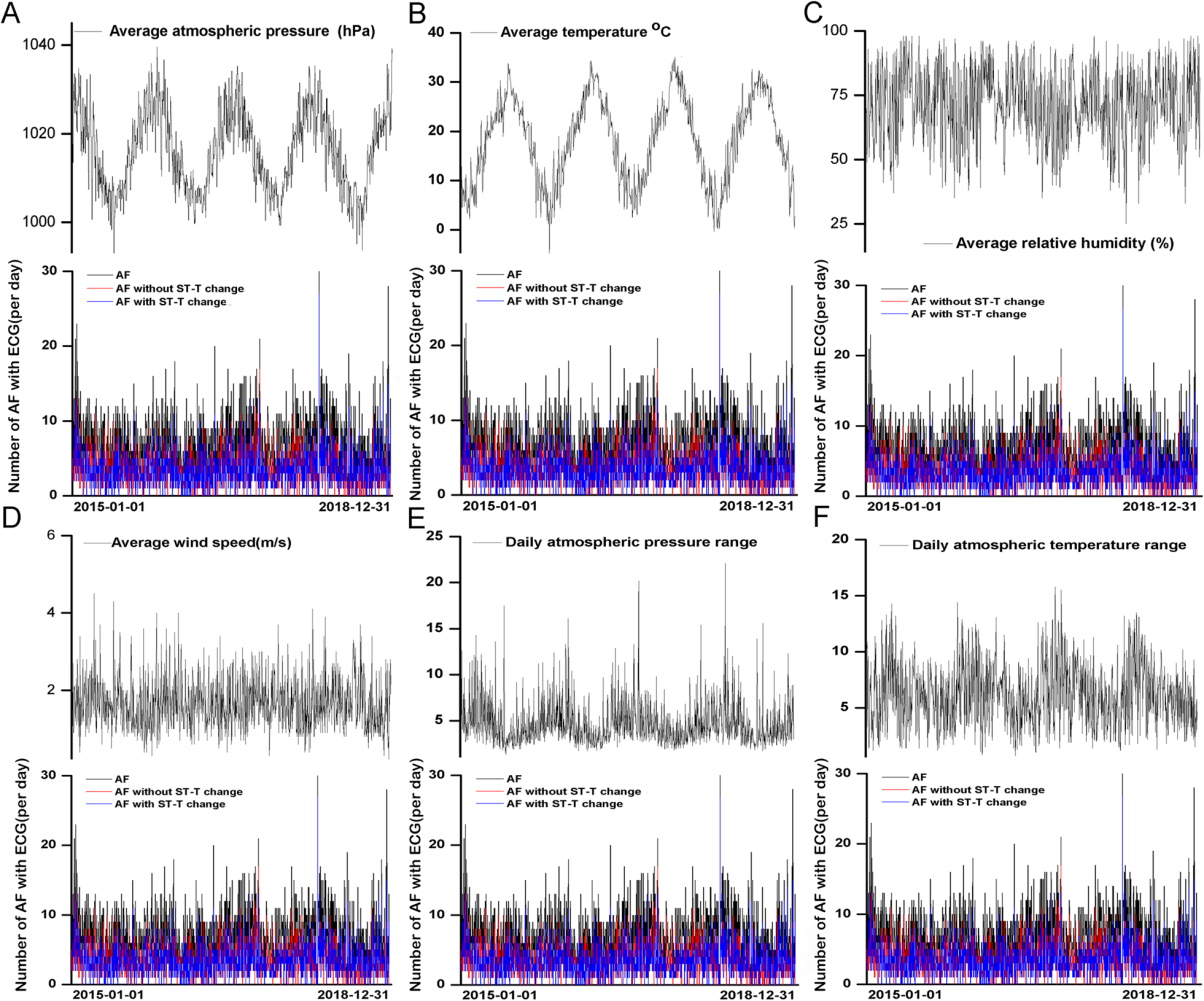
Time series diagram of meteorological factors (**A** average atmospheric pressure, **B** average temperature, **C** average relative humidity, **D** average wind speed, **E** daily atmospheric pressure range, **F** daily atmospheric temperature range) and atrial fibrillation cases

We recorded 10,325 cases of AF (Fig. 1). All patients eligible for the study exhibited AF indicated by ECG from January 1, 2015, to December 31, 2018. Exclusion criteria involved 538 repeated ECGs within a 48-h timeframe (Fig. 1). The final study cohort comprised 4933 male and 5392 female patients. After the correction, we observed associations between meteorological factors and hospital visits for AF patients with ECG. Among the AF patients with ST-T changes, the number of female group was 3156 (62.42%). Furthermore, the AF patients with ST-T changes tended to be older compared to those without ST-T changes (Table 1). The distribution of AF cases with ST-T segment changes across days is illustrated in Fig. 2.

### The relationship of meteorological factors and the air pollution

Due to the observed correlation between air pollutants and meteorological factors, necessary corrections were applied to the data analysis. As outlined in Additional file 1: Table S1, measurements were taken for particulate matter, gaseous air pollutants, and various meteorological variables. The analysis was adjusted using the data from air pollutant measurements to more accurately determine the impact of meteorological factors on hospital visits by patients with AF.

For the period from January to December 2015–2018, Spearman’s Rho-related technology was utilized to examine the correlation between meteorological variables and air pollutants (Additional file 1: Table S1). The results of Spearman’s rho correlation indicated positive associations among various air pollutants. With the exception of average temperature, average relative humidity, and average wind speed, which showed no significant correlation with the number of AF patients, all other factors were found to be related. This correlation analysis revealed a significant relationship between meteorological factors and air pollution, with further details provided in Additional file 1: Table S1. Refer to Additional file 1: Table S1 for comprehensive information on the correlations among meteorological variables and air pollutants.

### The variations in hospital visits for symptomatic AF patients with ECG

Based on the analysis of the data in Fig. 2, the time-series chart depicts the changing trends of meteorological variables over time. Most of the meteorological factors display distinct periodic variations, and the overall trends remain relatively stable. Notably, temperature and air pressure exhibit evident seasonal patterns. The variations in hospital visits for AF patients across different quarters are likely influenced by these meteorological factors.

Upon analyzing the data from Fig. 2, we observed a significant seasonal fluctuation in the number of AF patients visiting the hospital (*P* < 0.05). The lowest mean number of events was recorded in the third quarter compared to the other quarters (569 ± 54.75). Conversely, the first quarter showed the highest number of AF cases (744 ± 57.10), which data not shown.

### Partial meteorological factors have relationship with daily hospital visits of AF with ECG

The lag analysis method was used to explore the correlation between meteorological factors and AF occurrence, taking into account the lag effect on PM [26]. Conditional logistic regression (R software “season” package) was applied to analyze the effects of various lag periods of average atmospheric pressure, average temperature, average relative humidity, average wind speed, daily atmospheric pressure range, and daily atmospheric temperature range on daily AF cases, while correcting for pollutants (PM_2.5_, PM_10_, O_3_, SO_2_, NO_2_, and CO).

**Fig. 3:**
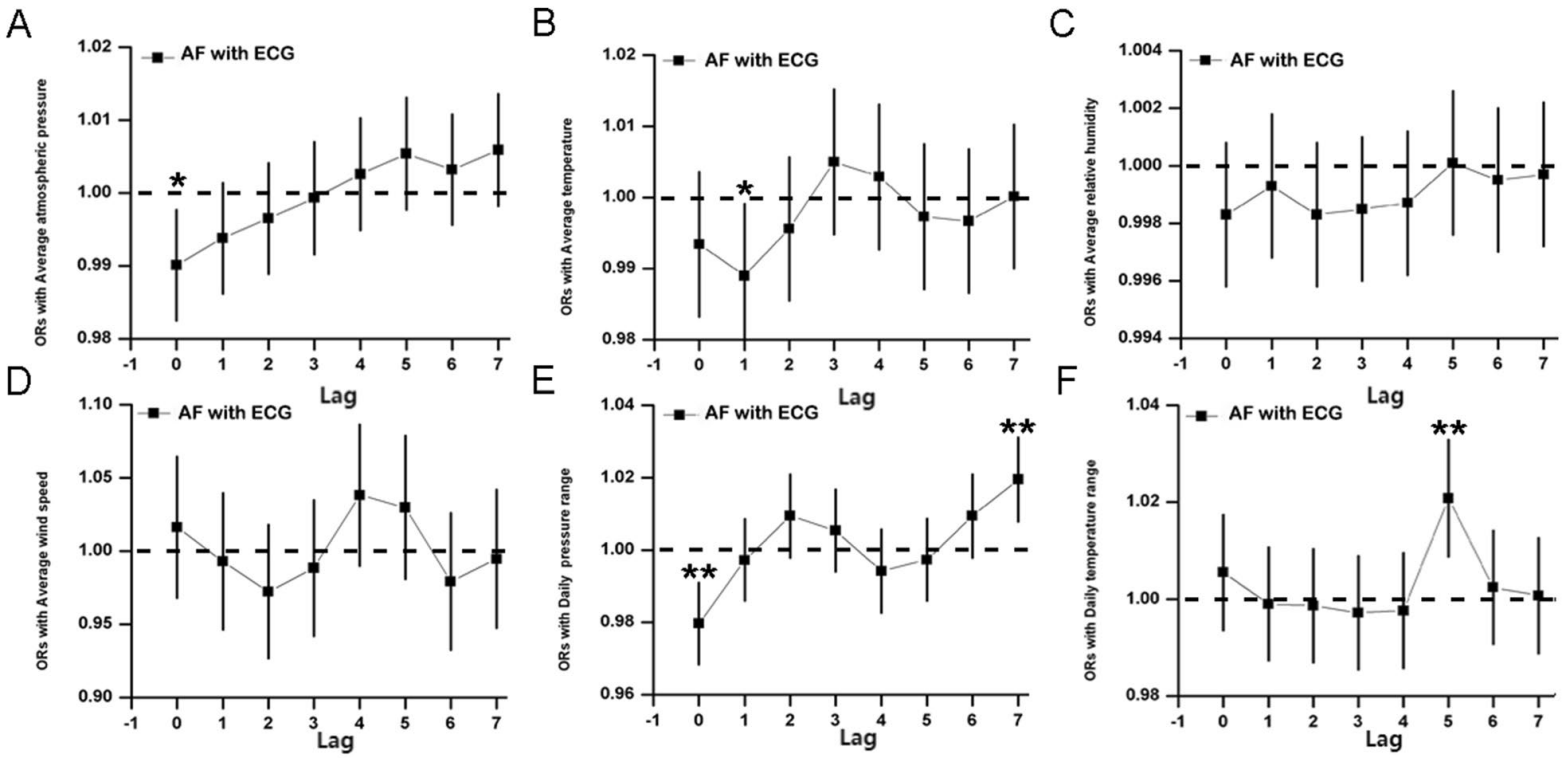
Lag analysis chart of meteorological factors on the hospital visits of atrial fibrillation after correcting air pollution. **A** The ORs with average atmospheric pressure, **B** ORs with average temperature, **C** the ORs with average relative humidity, **D** ORs with average wind speed, **E** ORs with daily pressure range, **F** ORs with daily temperature range. *P* value of less than 0.05 is considered to be statistically significant, which is represented by *; *P* value of less than 0.01 is considered to be more statistically significant, which is represented by **

**Fig. 4:**
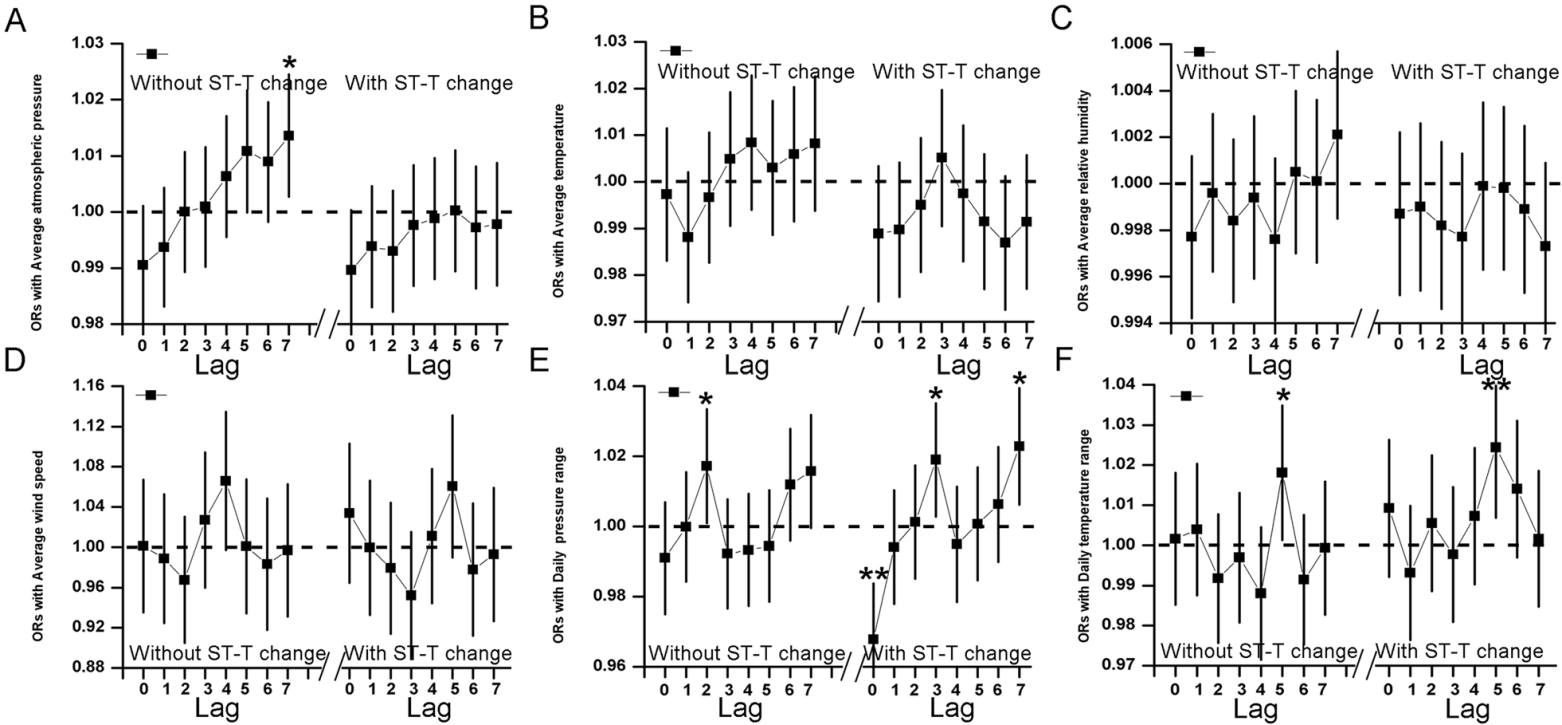
Lag analysis chart of meteorological factors on the disease of atrial fibrillation subgroup after correcting air pollution. **A** The ORs with average atmospheric pressure, **B** ORs with average temperature, **C** the ORs with average relative humidity, **D** ORs with average wind speed, **E** ORs with daily pressure range, **F** ORs with daily temperature range. *P* value of less than 0.05 is considered to be statistically significant, which is represented by *; *P* value of less than 0.01 is considered to be more statistically significant, which is represented by **

Upon correcting the relevant pollutant data (PM_2.5_ daily mean, PM_10_ mean, O_3_-day maximum 8-h mean, SO_2_ mean, NO_2_ mean, CO daily mean), the results revealed that average atmospheric pressure and average temperature were protective factors (lag 0: OR 0.9901, 95% CI 0.9825–0.9977, *P* < 0.05, and lag 1: OR 0.9890, 95% CI 0.9789–0.9992, *P* < 0.05) (Fig. 3AB). Conversely, a statistically significant correlation was observed between AF with constant ST-T segment and average atmospheric pressure 7 days before the event (lag 7: OR 1.0136, 95% CI 1.0027–1.0246, *P* < 0.05) (Fig. 4A). This indicates that average atmospheric pressure acts as a risk factor for AF patients with ST-T changes, while no significant correlation was found between AF with ST-T segment changes and average atmospheric pressure.

The analysis further revealed a statistically significant correlation between daily pressure range and AF events, with the association fluctuating over time. Initially, it acted as a protective factor (lag 0: 95% CI 0.9684–0.9913, *P* < 0.01) (Fig. 3E), but became a risk factor after 7 days (lag 7: OR 1.019, 95% CI 1.0079–1.0312, *P* < 0.01) (Fig. 3E). In subgroup analysis, the daily pressure range was a risk factor for patients with no ST-T segment change (lag 2: OR 1.0172, 95% CI 1.0009–1.0337, *P* < 0.05), while AF with ST-T changes exhibited positive correlations with daily pressure range at various lag periods (lag 0: OR 0.9734, 95% CI 0.9592–0.9878, *P* < 0.01; lag 3: OR 1.0162, 95% CI 1.0021–1.0306, *P* < 0.05; lag 7: OR 1.0211, 95% CI *P* < 0.05) (Fig. 4E), indicating heightened vulnerability. Hence, daily atmospheric pressure range is considered a risk factor for AF events.

Moreover, daily atmospheric temperature range was identified as a risk factor (lag 5: OR 1.0208, 95% CI 1.0087–1.0331, *P* < 0.01) (Fig. 3F). In the subgroup analysis, patients with no ST-T segment changes also showed a correlation with daily temperature range (lag 5: OR 1.0181, 95% CI 1.0013–1.0351, *P* < 0.05), while patients with ST-T changes displayed a more pronounced correlation (lag 5: OR 1.0244, 95% CI 1.0069–1.0422, *P* < 0.01) (Fig. 4F), indicating increased vulnerability. Conversely, no significant differences were found between average relative humidity, average wind speed, and AF events (Fig. 3C, D).

In contrast to previous literature, this study divided the population into subgroups based on ECG ST-T segment changes, an aspect not previously explored. In the subgroup analysis, for patients with no ST-T segment changes, only average atmospheric pressure displayed an influencing lag factor, while for patients with ST-T segment changes, the lag factor aligned with the overall correlation. This subgroup analysis suggests that patients with ST-T segment changes are more susceptible to daily atmospheric pressure range and daily atmospheric temperature range. For further data display, the relationship between meteorological factors and uncorrected pollutants’ effects on AF was analyzed and can be found in the attachment. We removed the air pollutant variable from the main analytical model, and then we used a distributed lag nonlinear model (DLNM) to capture the non-linear relation between the meteorological factors and the frequency of AF. Their trends are consistent with the results of the main analysis (Additional files 1, 2 and 3: Figs. S1–S3).

## Discussion

### Main findings

The present study aimed to investigate the impact of meteorological factors on AF in a subtropical monsoon climate. Our findings indicate a significant correlation between meteorological factors and AF occurrence. In specific, average atmospheric pressure, daily atmospheric pressure range, and daily atmospheric temperature range showed notable associations with AF events with ECG. Notably, patients with ST-T segment changes exhibited a heightened susceptibility to meteorological factors, resulting in a higher overall number of hospital visits for AF with ECG. These results suggest that meteorological factors may contribute to an increased prevalence of AF with ST-T segment changes.

### Meteorological factors are related to the increase of AFs

This study demonstrates a significant correlation between AF and meteorological factors, particularly average atmospheric pressure, daily atmospheric pressure range, and daily atmospheric temperature range. Our findings align with previous research conducted by other scholars in this field. A preliminary study showed that meteorological factors are associated with an increased incidence of AF, with the seasonal occurrence being highest in winter and lowest in summer. The study also highlighted that cold temperatures increased the risk of cardiovascular disease, while humidity played a role in increasing demand for cardiovascular disease ambulances during warmer seasons. Thus, cardiovascular diseases are more likely to occur in cold weather and in high humidity conditions. These findings are consistent with other studies that have explored the relationship between temperature, humidity, and cardiovascular diseases [27–30].

In our research, we also established the relationship between AF with ECG and meteorological factors, especially average atmospheric pressure, daily atmospheric pressure range, and daily atmospheric temperature range. The higher the daily pressure range and daily temperature range, the greater the number of AF patients with ECG observed. However, we did not find a positive correlation between humidity and AF in our data. In addition, a novel aspect of our study is the comprehensive investigation of AF, taking into account both ST-T segment changes and ST-T segment unchanged cases by analyzing ECG records. This is the first study of patients with AF combined with ST-T changes.

Based on our research, we recommend that patients with AF and ST-T changes should be particularly mindful of meteorological factors, especially average atmospheric pressure, daily pressure range, and daily temperature range. Taking preventive measures to minimize temperature differences in weather conditions with large fluctuations may help reduce the risk of disease. While individuals cannot control long-term environmental changes such as air pressure fluctuations, considering control their home temperature might be an option for some.

Overall, our study sheds light on the relationship between meteorological factors and AF, specifically for patients with ST-T changes, offering valuable insights into potential risk management strategies for individuals susceptible to weather-related influences.

### The mechanism of meteorological factors associated with arrhythmia

To date, the exact biological mechanisms through which meteorological factors induce arrhythmia remain incompletely understood. Current research suggests that air pollutants might cause acute cardiac events by affecting cardiac autonomic function, myocardial repolarization, local and systemic inflammation, reactive oxygen species, coagulation, and myocardial ischemia [31]. The development of AF involves three main mechanisms: inflammation, oxidative stress, and the autonomic nervous system [32–35]. In addition, the Ang-II-MAPK and TGF-β1-Smad signaling pathways are thought to play central roles in regulating atrial fibrotic remodeling in AF. The balance between miRNA molecules exerting anti-/profibrotic effects is crucial for controlling atrial fibrosis in AF. Moreover, the interplay between cardiac matrix metalloproteinases (MMPs) and their endogenous tissue inhibitors is critical for atrial extracellular matrix (ECM) metabolism and fibrosis [36].

Furthermore, the K_2_P channel family, widely expressed in various human cell types and organs, regulates important physiological processes. Its activity is influenced by stimuli such as pH levels and temperature [37]. Meteorological factors have been shown to affect these inducers, suggesting that patients with ST-T segment changes are more susceptible to cardiovascular diseases, including AF, which aligns with our research data. Previous studies have demonstrated that outdoor temperature and air pressure may mediate monthly fluctuations in AF, with the highest number of hospital visits occurring in December and the lowest in June. Meteorological conditions impact the severity of AF and hospitalization rates [38]. In addition, the correlation between cardiovascular disease and meteorological factors has been investigated, revealing a linear positive correlation between air pressure and admissions for cardiovascular diseases, and a linear negative correlation between temperature and cardiovascular disease admissions [39].

However, specific mechanisms underlying these associations still require further study. Continued research is needed to gain a deeper understanding of how meteorological factors influence the occurrence and progression of cardiovascular diseases, including AF.

### The mechanism of ST-T changes of ECG

The focus of the following discussion is primarily on repolarization activity, particularly the ST-T changes associated with it. The cardiac repolarization process involves various phases, including a rapid but transient early repolarization phase (phase 1) and a relatively positive plateau phase (phase 2) lasting over 100 ms. During phase 2, inward currents like small components of INa and especially the L-type Ca^2+^ current balance outward currents. The ST segment represents a period of no potential change between the end of ventricular depolarization and the beginning of repolarization, signifying the slow repolarization of the ventricle. On the other hand, the T segment indicates the rapid repolarization of the ventricular muscle. The ST-T segment, mainly related to phase 2, represents the stage of ventricular repolarization [40].

In AF, increased heart rates lead to more Ca^2+^ entering the cell. Studies have observed a 60% reduction in Ito in cardiomyocytes from AF patients. In addition, alterations in inward-rectifier K^+^ currents occur in AF and might play a crucial role in repolarization changes and arrhythmogenesis [38]. Analyzing the ST-T changes in an electrocardiogram provides a comprehensive understanding of the cardiomyocyte repolarization vector. The 12-lead electrocardiogram effectively reflects the overall cardiac repolarization process. However, other cardiovascular diseases, such as myocardial ischemia, hypertension, cardiomyopathy, and electrolyte abnormalities, can also cause abnormal cardiomyocyte repolarization, leading to changes in the electrocardiogram ST-T segment. When AF is combined with these underlying diseases, ST-T changes often occur, indicating abnormal repolarization in the electrocardiogram. The presence of ST-T changes in patients with AF is often associated with more severe underlying diseases compared to patients without such changes. Therefore, the assessment of ST-T changes serves as a convenient means to classify the severity of underlying diseases in patients with AF [41]. Consequently, research on the changes in the ST-T segment is of paramount importance for studying AF and its implications.

### Study limitation

This study has several limitations that should be acknowledged. First, the data used in this study came from a single-center database, which may limit the comprehensiveness of the findings. It only includes AF patients with ECG who visited Shanghai Sixth People's Hospital, potentially excluding cases from other healthcare centers. Meanwhile, our main objective was to evaluate the short-term impact of meteorological factors on hospital visits for AF patients with detected ECG, but due to time constraints, we did not assess the long-term legacy effects of meteorological factors. Moreover, we did not investigate the changes in ventricular repolarization or QT intervals after exposure to corresponding meteorological factors, leaving the underlying mechanism linking meteorological factors and AF unclear. While we explored the relationship between meteorological factors and AF with ST-T changes, further research is needed to understand the broader impact on ventricular repolarization.

In addition, AF cases detected by 24-h ambulatory electrocardiogram were not included in the statistics due to the inability to evaluate 12-lead ST-T changes. This may have led to missing data on some patients with acute-onset AF who refused ECG or were not included in regular follow-up. It is important to consider that the time of onset of AF and the timing of seeking treatment can vary among individuals, and the duration of AF onset might influence ST-T segment changes [42, 43].

Despite these limitations, this study contributes to understanding the correlation between meteorological factors and AF with ST-T changes, shedding light on the potential impact of weather patterns on AF occurrence. Further multi-center studies with larger sample sizes and comprehensive assessment of repolarization changes are necessary to fully elucidate the mechanisms underlying this association.

## Conclusions

In conclusion, our study revealed a significant association between meteorological factors and the occurrence of AF in patients with ECG-detected ST-T changes visiting our hospital. In specific, we found a correlation between AF and average atmospheric pressure, daily pressure range, and daily temperature range. This association was more pronounced in patients with ST-T segment changes, indicating their heightened vulnerability to meteorological factors, which contributed to the overall increase in hospital visits for AF. Furthermore, we observed a positive correlation between meteorological factors and AF overall, suggesting a broader impact. However, the underlying pathophysiological mechanisms linking meteorological factors and AF require further investigation. Future research should delve deeper into understanding the intricacies of this relationship.

### Supplementary Information

**Additional file 1: Table S1.** Data of air pollution correction and meteorological factors. Statistical significance was considered three levels at *p* < 0.05, *p* < 0.01, and *p* < 0.001 to see the aggregative effect of each meteorological parameters, and the results show a correlation between pollutants and meteorological factors, but the correlation is not strong.

**Additional file 2: Figure S1.** The lag analysis of meteorological factors on the disease of atrial fibrillation subgroup without correcting air pollution. It can be seen from the figure that these six meteorological factors have different degrees of hysteresis. **A** shows the ORs with Average atmospheric pressure, **B** shows ORs with Average temperature, **C** shows the ORs with Average relative humidity, **D** shows ORs with Average wind speed, **E** shows ORs with daily pressure range, **F** shows ORs with daily temperature range. *P* value of less than 0.05 is considered to be statistically significant, which is represented by *; *P *value of less than 0.01 is considered to be more statistically significant, which is represented by **.

**Additional file 3: Figure S2.** The lag analysis of meteorological factors on the disease of atrial fibrillation subgroup without correcting air pollution. The hysteresis of patients with ST-T-segment changes can be seen by subgroup analysis was basically the same as the general trend. **A** shows the ORs with Average atmospheric pressure, **B** shows ORs with Average temperature, **C** shows the ORs with Average relative humidity, **D** shows ORs with Average wind speed, **E** shows ORs with daily pressure range, **F** shows ORs with daily temperature range. *P* value of less than 0.05 is considered to be statistically significant, which is represented by *; *P* value of less than 0.01 is considered to be more statistically significant, which is represented by **.

**Additional file 4: Figure S3.** Associations from distributed lag nonlinear models of daily meteorological factors with the risk of atrial fibrillation. Relative risks (RRs) and 95% confidence intervals (CIs) of atrial fibrillation were calculated for **A** average atmospheric pressure over 0–7 lag days, **B** average temperature over 0–7 lag days, **C** average relative humidity over 0–7 lag days, **D** average wind speed over 0–7 lag days, **E** daily pressure range over 0–7 lag days, **F** daily temperature range over 0–7 lag days.

## Data Availability

The dataset supporting the conclusions of this article is included within the article (and its additional file).
