# Supplementary figures and images for: Rapid weather changes are associated with daily hospital visitors for atrial fibrillation accompanied by abnormal ECG repolarization: a case-crossover study
Source: Eur J Med Res. 2024 Jan 20;29:62. doi: 10.1186/s40001-023-01632-3 (PMC10799445; doi:10.1186/s40001-023-01632-3)

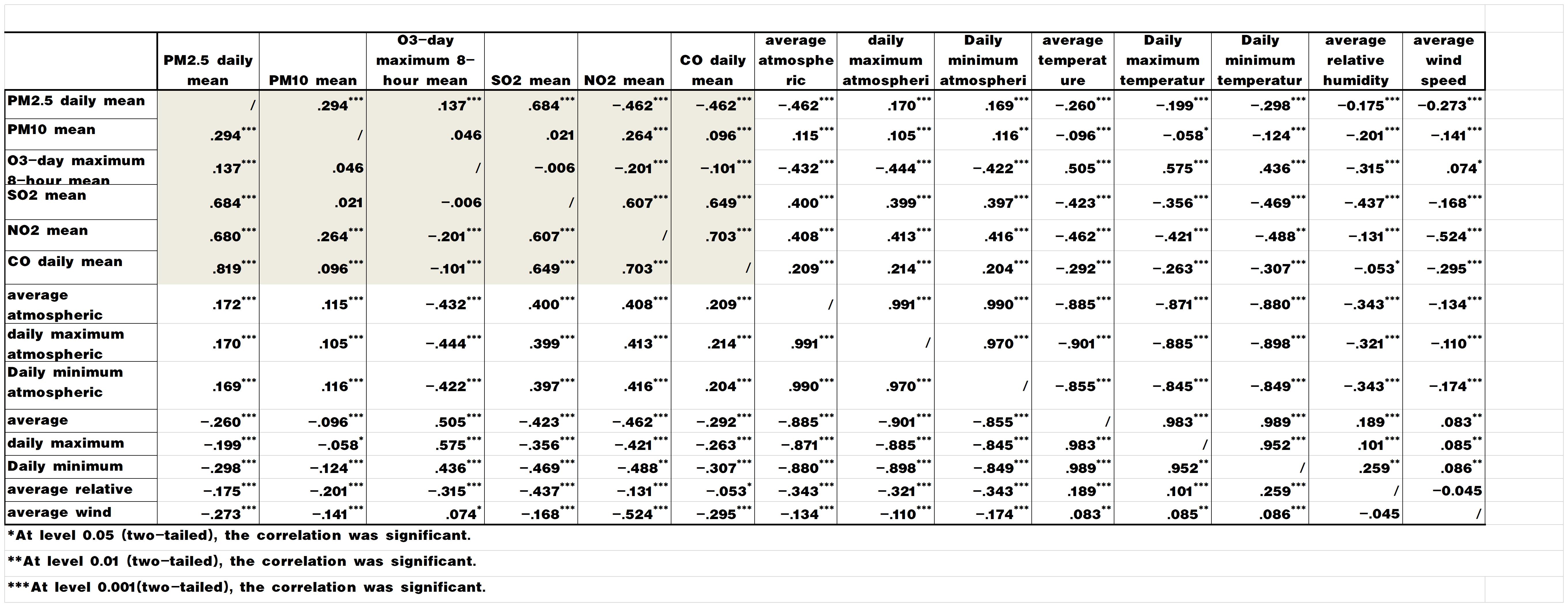

Supplement: Supplementary file 1 — Additional file 1: Table S1. Data of air pollution correction and meteorological factors. Statistical significance was considered three levels at p < 0.05, p < 0.01, and p < 0.001 to see the aggregative effect of each meteorological parameters, and the results show a correlation between pollutants and meteorological factors, but the correlation is not strong. [file 40001_2023_1632_MOESM1_ESM.jpg]

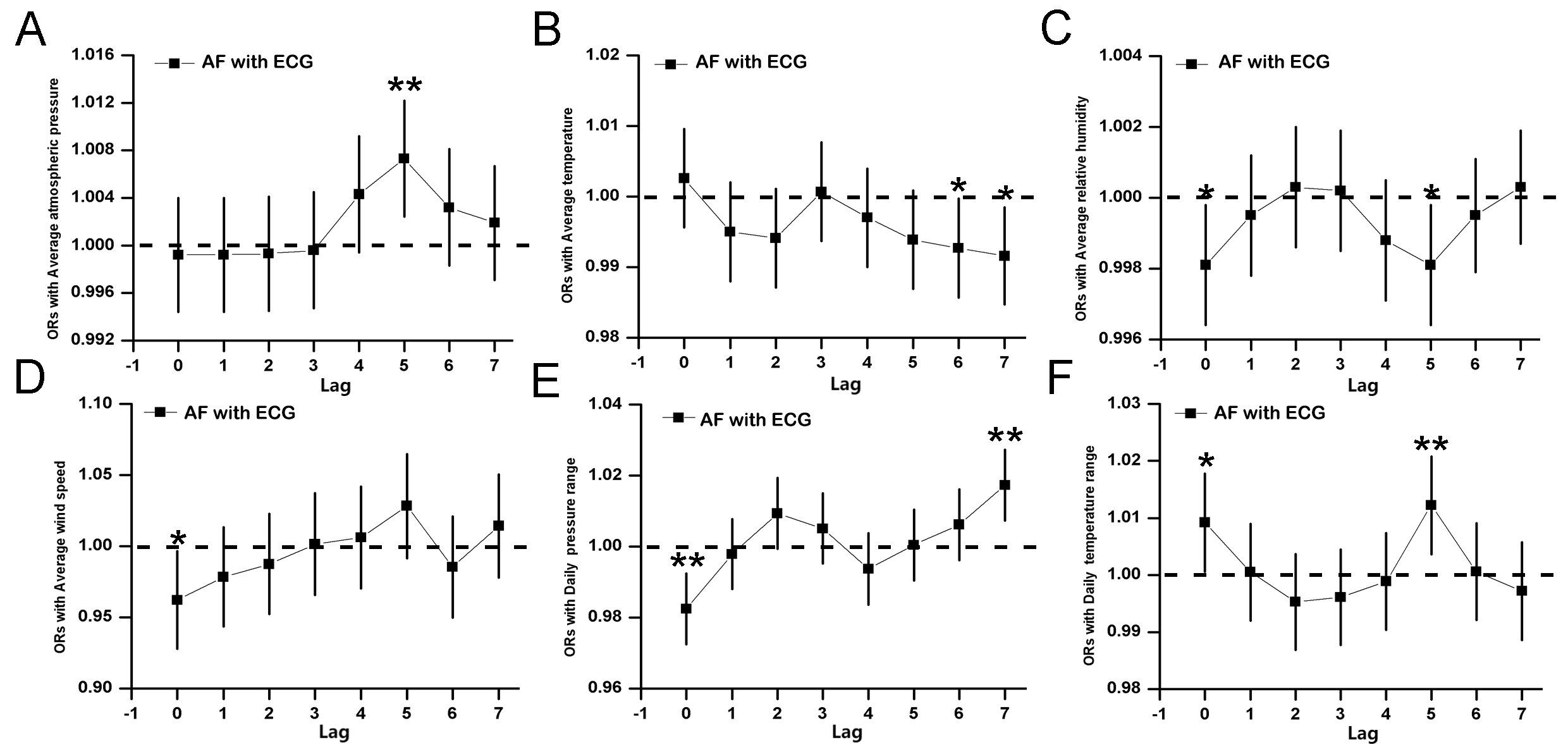

Supplement: Supplementary file 2 — Additional file 2: Figure S1. The lag analysis of meteorological factors on the disease of atrial fibrillation subgroup without correcting air pollution. It can be seen from the figure that these six meteorological factors have different degrees of hysteresis. A shows the ORs with Average atmospheric pressure, B shows ORs with Average temperature, C shows the ORs with Average relative humidity, D shows ORs with Average wind speed, E shows ORs with daily pressure range, F shows ORs with daily temperature range. P value of less than 0.05 is considered to be statistically significant, which is represented by *; P value of less than 0.01 is considered to be more statistically significant, which is represented by **. [file 40001_2023_1632_MOESM2_ESM.png]

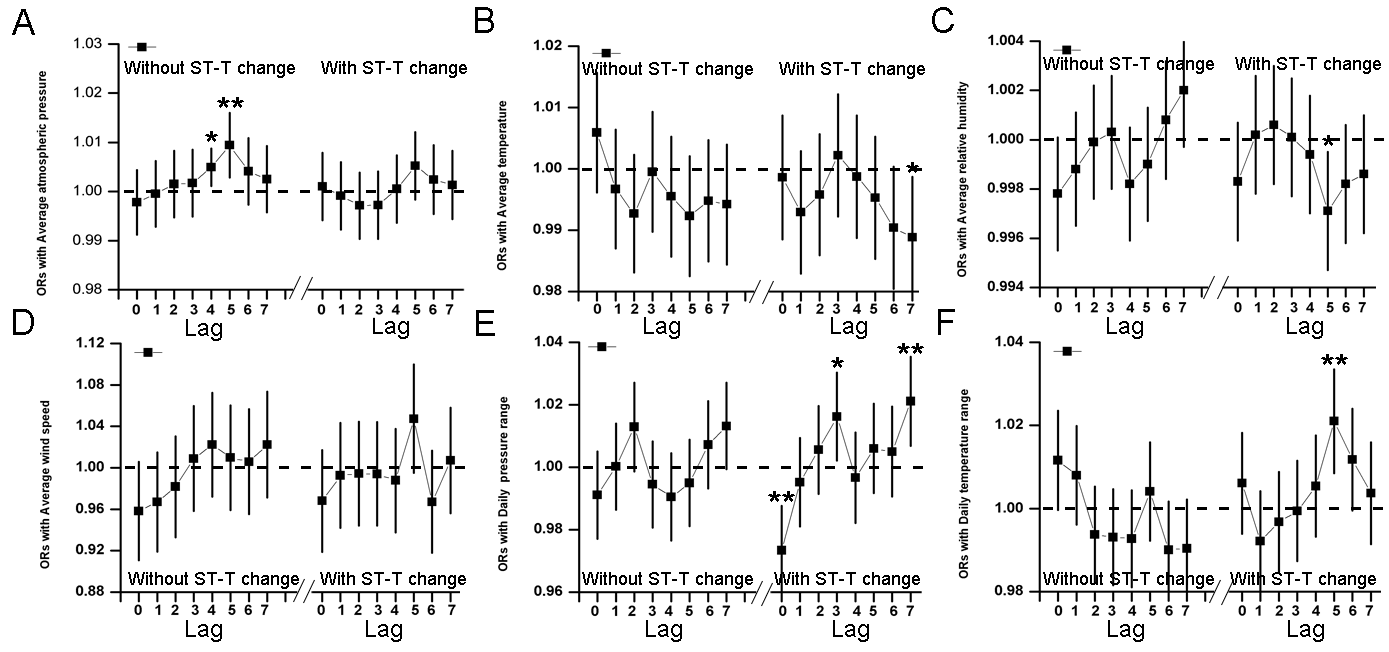

Supplement: Supplementary file 3 — Additional file 3: Figure S2. The lag analysis of meteorological factors on the disease of atrial fibrillation subgroup without correcting air pollution. The hysteresis of patients with ST-T-segment changes can be seen by subgroup analysis was basically the same as the general trend. A shows the ORs with Average atmospheric pressure, B shows ORs with Average temperature, C shows the ORs with Average relative humidity, D shows ORs with Average wind speed, E shows ORs with daily pressure range, F shows ORs with daily temperature range. P value of less than 0.05 is considered to be statistically significant, which is represented by *; P value of less than 0.01 is considered to be more statistically significant, which is represented by **. [file 40001_2023_1632_MOESM3_ESM.png]

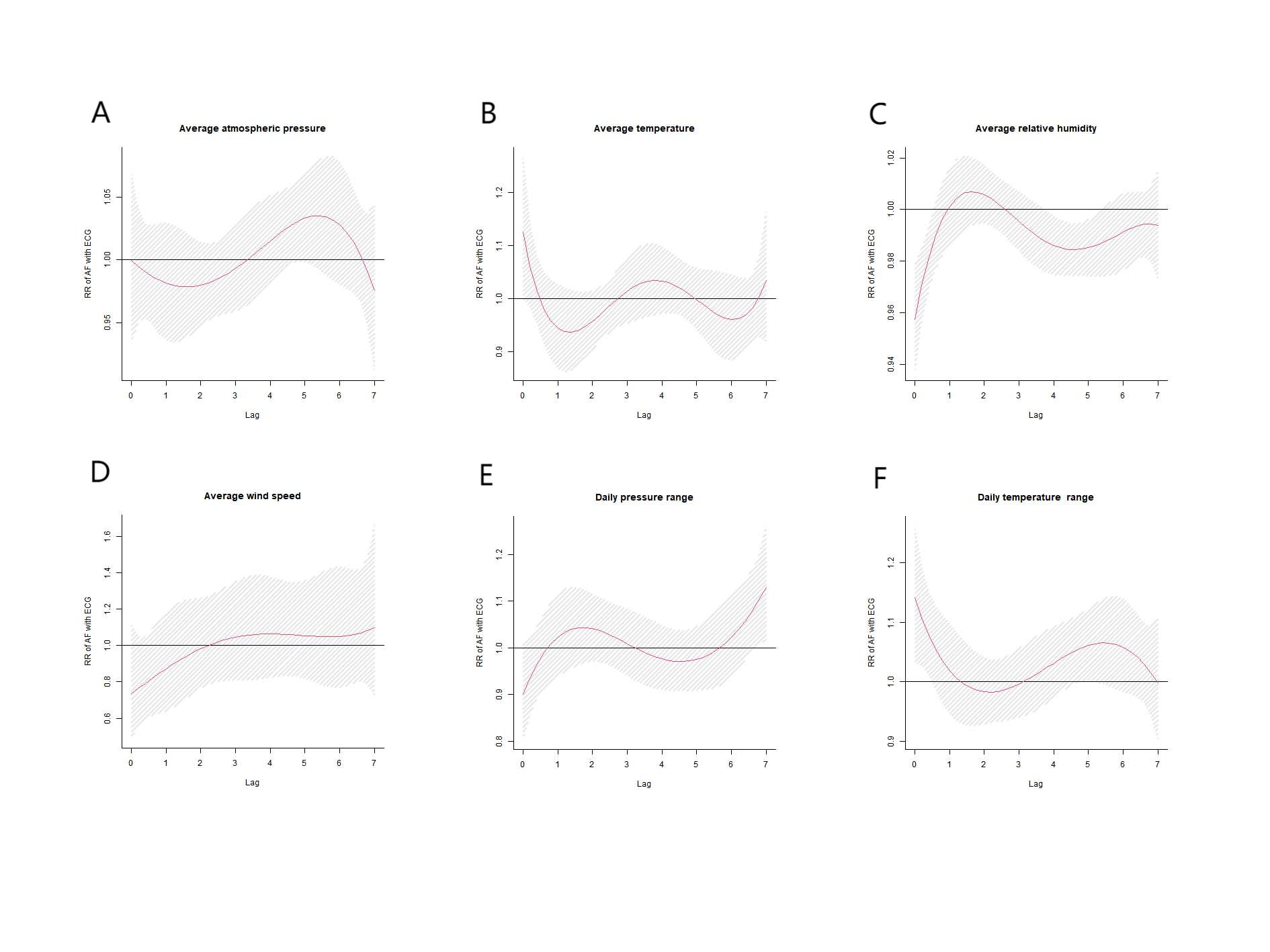

Supplement: Supplementary file 4 — Additional file 4: Figure S3. Associations from distributed lag nonlinear models of daily meteorological factors with the risk of atrial fibrillation. Relative risks (RRs) and 95% confidence intervals (CIs) of atrial fibrillation were calculated for A average atmospheric pressure over 0–7 lag days, B average temperature over 0–7 lag days, C average relative humidity over 0–7 lag days, D average wind speed over 0–7 lag days, E daily pressure range over 0–7 lag days, F daily temperature range over 0–7 lag days. [file 40001_2023_1632_MOESM4_ESM.png]
